# Methods to Monitor and Quantify Autophagy in the Social Amoeba *Dictyostelium discoideum*

**DOI:** 10.3390/cells6030018

**Published:** 2017-07-03

**Authors:** Eunice Domínguez-Martín, Elena Cardenal-Muñoz, Jason S. King, Thierry Soldati, Roberto Coria, Ricardo Escalante

**Affiliations:** 1 Instituto de Investigaciones Biomédicas “Alberto Sols” (CSIC-UAM), Arturo Duperier 4, 28029 Madrid, Spain; edominguez@iib.uam.es; 2 Departamento de Genética Molecular, Instituto de Fisiología Celular, Universidad Nacional Autónoma de México, 04510 Ciudad de México, Mexico; rcoria@ifc.unam.mx; 3 Département de Biochimie, Faculté des Sciences, Université de Genève, Sciences II, CH-1211-Genève-4, Switzerland; Elena.Cardenal@unige.ch (E.C.-M.); Thierry.Soldati@unige.ch (T.S.); 4 Department of Biomedical Sciences and Bateson Centre, University of Sheffield, Sheffield S10 2TN, UK; jason.king@sheffield.ac.uk

**Keywords:** autophagy, *Dictyostelium*, flux assays, autophagic markers, cleavage assays

## Abstract

Autophagy is a eukaryotic catabolic pathway that degrades and recycles cellular components to maintain homeostasis. It can target protein aggregates, superfluous biomolecular complexes, dysfunctional and damaged organelles, as well as pathogenic intracellular microbes. Autophagy is a dynamic process in which the different stages from initiation to final degradation of cargo are finely regulated. Therefore, the study of this process requires the use of a palette of techniques, which are continuously evolving and whose interpretation is not trivial. Here, we present the social amoeba *Dictyostelium discoideum* as a relevant model to study autophagy. Several methods have been developed based on the tracking and observation of autophagosomes by microscopy, analysis of changes in expression of autophagy genes and proteins, and examination of the autophagic flux with various techniques. In this review, we discuss the pros and cons of the currently available techniques to assess autophagy in this organism.

## 1. Introducing *Dictyostelium* as a Model for Autophagy Research

Intracellular degradation processes are ubiquitous to all organisms and essential to maintain cellular homeostasis. One of the major degradative pathways in eukaryotes is macroautophagy (hereafter referred to as autophagy), a dynamic process that captures and delivers diverse cellular material, including cytoplasm, organelles, and protein aggregates, to the lysosomes. Although basal levels of autophagy might be sufficient for homeostasis under normal growth conditions, a variety of stresses induce a strong up-regulation of this pathway, such as starvation [1], mechanical deformations [2], oxidative and endoplasmic reticulum (ER) stresses [3], and infection [4], among others. In order to reach the degradation stage, the cargo must be sequestered from the cytosol by de novo formation of a double-membrane cisterna, the phagophore. In yeast, this occurs only at one site near the vacuole, known as the phagophore assembly site (PAS). In other eukaryotes, including *Dictyostelium discoideum* and human cells, this occurs at multiple sites on the ER, and the initial structure formed is referred to as the omegasome. The phagophore expands using membranes from many sources, such as the ER, Golgi, endosomes, mitochondria, and plasma membrane [5,6], eventually closing and generating a completed vesicle, the autophagosome.

Although the study of autophagy dates back to the early 1960s, when C. de Duve coined the term [7], it is still a growing research field, mainly due to its complexity and importance in many physiological and pathological aspects of human health and disease [8]. A large number of proteins that participate in and regulate the autophagic process have been defined (termed Atg proteins). Many of these studies have been performed in model organisms, mainly yeast, as recognized recently by the Nobel prize awarded to Yoshinori Ohsumi for his discoveries on the autophagic machinery [9,10].

One of the organisms that has recently contributed to this area is *Dictyostelium*, a soil amoeba that was isolated and described by Kenneth Raper in 1935. Since then, it has become a well-established model in cell and developmental biology [11,12]. Importantly, *Dictyostelium* has conserved genes involved in processes that were lost during the specialization of fungi, such as phagocytosis, macropinocytosis, chemotaxis, and motility. So, in many ways (including autophagosome formation), *Dictyostelium* is more similar to metazoans than yeasts are [13], justifying the use of *Dictyostelium* as a model for human diseases and even for drug screening [14,15,16,17,18,19,20]. For example, research on *Dictyostelium* has contributed to the unraveling of the signaling pathways implicated in the poorly defined therapeutic effects of valproic acid and lithium [18]. Additionally, *Dictyostelium* has proven to be a good model organism to address questions regarding the etiology of some human diseases; for instance, orthologues of the genes implicated in neurological disorders such as neuronal ceroid lipofuscinosis [21], chorea-acanthocytosis [22], Alzheimer’s disease [23], and lissencephaly [24] (among others) have been studied in *Dictyostelium*, shedding light on the cellular biology underlying these conditions. Further, as many innate immunity mechanisms used by animal phagocytes are conserved in *Dictyostelium*, studies using this amoeba as a model organism have expanded the knowledge on the molecular mechanisms of infection and pathogen–host interactions [20,25].

**Figure 1:**
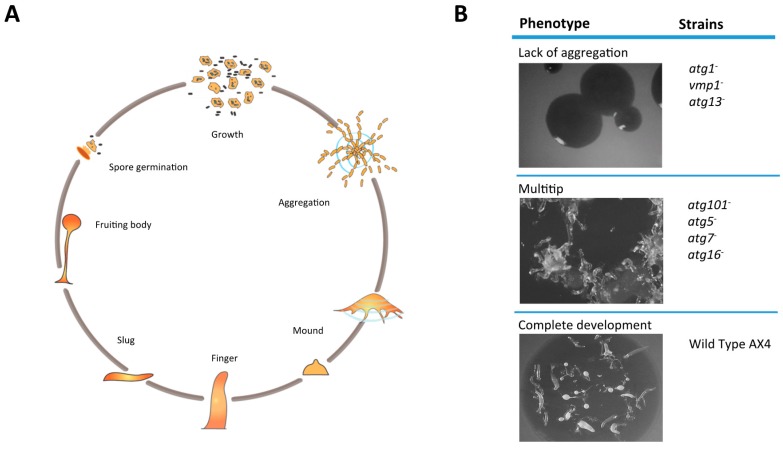
*Dictyostelium* as a model for autophagy. *Dictyostelium discoideum* is a social amoeba that enters a developmental program during starvation. (**A**) Scheme of the *Dictyostelium* developmental cycle. Individual amoebas aggregate to form mounds of cells that undergo different stages of development that culminate with the formation of a fruiting body containing spores. (**B**) The lack of autophagy in *Dictyostelium* leads to developmental arrest either at the aggregation stage or at the mound stage. In the latter, the formation of multiple tips is characteristic of some of the strains.

*Dictyostelium* has an unusual life cycle that alternates between unicellular and multicellular phases. As a unicellular amoeba, *Dictyostelium* feeds on bacteria and yeasts and multiplies by fission about every 8 h. However, when nutrients are scarce, vegetative growing cells enter a developmental program that begins with the secretion of diverse molecules for intercellular communication. One of these, cyclic adenosine monophosphate (cAMP), functions as a chemoattractant, resulting in cell aggregation. Collectives of about 100,000 cells then differentiate to form a multicellular slug that finally culminates in a fruiting body, a structure composed of a stalk of dead cells supporting a sorocarp filled with spores that will disperse and germinate under favorable environmental conditions (Figure 1A) (a comprehensive review on *Dictyostelium* development can be found in [26]). Interestingly, *Dictyostelium* defends itself against pathogens both as a single cell and as a multicellular organism. Inside the slug, a subset of specialized phagocytic cells called Sentinel cells function as a primitive innate immune system and patrol the facultative multicellular organism to scavenge and kill invading microbes by phagocytosis or using extracellular DNA traps [27,28].

During development, *Dictyostelium* cells rely on autophagy to obtain the energy and metabolites required for aggregation and differentiation [29]. However, besides this recycling function, autophagy also functionally participates in the developmental program. For example, autophagy is required for the vacuolization that accompanies differentiation and death of the stalk cells [30], and is required for the unconventional secretion of AcbA—the precursor of SDF-2, a protein involved in spore formation [31]. Consequently, a blockade in autophagy results in abnormal development.

Since the first *Dictyostelium* autophagy mutants were described [29,32], it became clear that the severity of the phenotypes correlates with the level of autophagy inhibition [33]. These phenotypes range from a complete lack of aggregation to incomplete development, characterized by the formation of multi-tipped structures and aberrant fruiting bodies, which has facilitated the isolation and characterization of many autophagy mutants (Figure 1B). Comprehensive reviews on autophagy in *Dictyostelium* can be found in [34,35]. It must be noted that the severity of the multi-tipped phenotype depends on the genetic background of the laboratory strain used. For example, while mutants of Atg16 in the AX4-background produce large, abnormal mounds with many finger-like tips [22], a similar mutant in AX2 forms smaller mounds with only three tips [36]. In both cases, however, as development progresses, the multi-tipped structures from both strains form highly abnormal fruiting bodies and few viable spores.

## 2. Strategies to Induce Autophagy in *Dictyostelium*

Starvation is the most common stimulus used to activate *Dictyostelium* autophagy in the laboratory. Like in other organisms, autophagy can be triggered by a complete starvation, which is accomplished by incubating the cells in a phosphate buffer, or by depleting lysine and arginine—two essential amino acids—and nitrogen sources from otherwise complete medium. Note that the two conditions vary in their impact on cell physiology, whilst the depletion of specific amino acids simply causes growth arrest and a loss of viability over time, a complete lack of nutrients additionally activates the developmental program. Usually, after about 15 min of either of the above treatments, the average number of autophagosomes increases from about 1 to around 2.5 per cell [2,37].

Mechanical stress also activates autophagy in *Dictyostelium* similarly to other eukaryotic cells. This can be accomplished by overlaying the cells with a thin agarose sheet [2]. In addition, it has been shown that the exposure to the *Staphylococcus aureus* lipopolysaccharide or the damage to the phagosomal membrane caused by *Mycobacterium marinum* infection induce an autophagic response [38,39].

**Figure 2:**
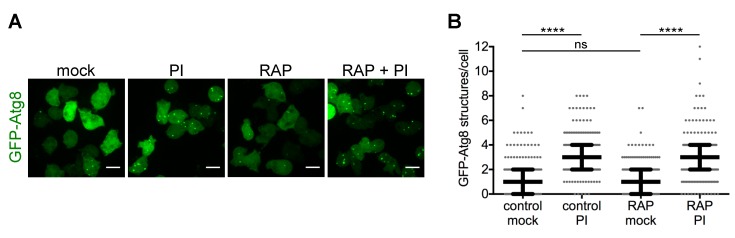
A short-term rapamycin (RAP) treatment does not induce autophagic flux in *Dictyostelium*. *Dictyostelium* Ax2(Ka) cells expressing green fluorescent protein (GFP)-Atg8 were treated or mock-treated with RAP at 500 nM for 2 h. One hour before the end of the treatment, cells were incubated or not with a protease inhibitor cocktail (PI, Roche 11873580001) at 2.5×. (**A**) Representative maximum projections of live cells under the treatments described above. Scale bars, 10 µm; (**B**) Median and interquartile ranges of the number of GFP-Atg8 structures per cell during the mentioned treatments. Each dot represents one cell; 162–168 cells per condition were counted. The values of λ that define the Poisson distribution of each data set and differences between them were calculated as described before [34] (**** *p* ≤ 0.0001; ns, *p* > 0.05). No significant differences were observed by quantification of the percentage of cells with GFP-Atg8 dots under RAP treatment (not shown).

In yeast and mammalian cells, rapamycin (RAP) is often used as an autophagy activator, which acts by allosterically inhibiting the mechanistic target of rapamycin complex 1 (mTORC1), which directly phosphorylates Atg1 and connects nutrient availability to autophagy induction. The effect of RAP on autophagy activation is therefore very rapid in most cell types (from minutes to a few hours), but it may not inhibit TOR completely. Other inhibitors such as Torin1, PP242, KU-0063794, PI-103, and NVP-BEZ235 target the catalytic domain of TOR and are more potent inhibitors, although they target both TORC1 and TORC2. In *Dictyostelium*, short-term RAP treatment does not seem to induce autophagy (Figure 2), despite reports showing that the functional TORC1 complex is sensitive to RAP inhibition [40]. Either the level of inhibition of TORC1 is insufficient to activate autophagy, or *Dictyostelium* has evolved other pathways to transmit the signal of nutrient availability to the autophagy machinery. However, long-term RAP treatment (24 h) has been reported to activate autophagy in *Dictyostelium* [41]. As these long treatments may have additional indirect effects, it is not clear whether the observed induction is the result of secondary cell damage instead of a direct signaling pathway. Further studies are required to unambiguously determine whether in *Dictyostelium* TORC1 directly signals to the Atg1 complex to regulate autophagy, as described in other organisms.

In contrast, the drug AR-12 (OSU-03012) has recently been shown to induce the autophagic flux in *Dictyostelium* [39]. A two-hour treatment with this drug—which induces autophagosome formation in mammals by various mechanisms, such as the inhibition of the 3-phosphoinositide-dependent protein kinase 1 (PDK1), the induction of ER stress, and the accumulation of reactive oxygen species (ROS) [42,43]—is enough to increase the number of autophagosomes and the rate of degradation of the GFP cleaved from GFP-Atg8 in *Dictyostelium* [39].

## 3. Microscopy Techniques

### 3.1. Transmission Electron Microscopy (TEM)

**Figure 3:**
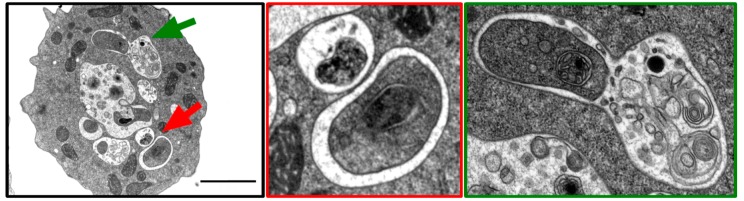
Visualization of autophagosomes in *Dictyostelium* by TEM. Electron micrographs of a *Dictyostelium* Ax2(Ka) cell treated with the autophagy inducer drug AR-12 at 2.5 µM for 2 h and with PI at 2.5× for 1 h. For imaging, cells were fixed for 1 h with 2% glutaraldehyde and stained for 30 min with a 2% osmium/0.1 M imidazole solution. Fixed cells were pelleted, washed in phosphate-buffered saline (PBS), and further processed, as previously described [38]. The red arrow marks the enlarged panel showing two autophagosomes; the green arrow marks the enlargement where a fusion event between a late endosome and an autophagosome can be observed. Scale bar, 2 µm.

TEM has been a key technique in the study of autophagy since its discovery. The first autophagic structures in *Dictyostelium* were observed using this method [44]. Additionally, the first studies that identified orthologous *atg* genes in this amoeba relied on TEM to evaluate autophagosome formation [29]. More recently, thanks to an improved fixation protocol, the double-membrane autophagic structures can be visualized with an unprecedented level of detail (Figure 3) [39,45]. However, due to the low number of autophagosomes, it is rather difficult to identify them in thin EM sections. Consequently, this technique remains essential to analyze the ultrastructure of abnormal autophagic organelles and the lack of bona fide autophagosomes [46], but is not adequate to quantitatively monitor the generation and fate of autophagic bodies.

### 3.2. Fluorescence Microscopy

**Figure 4:**
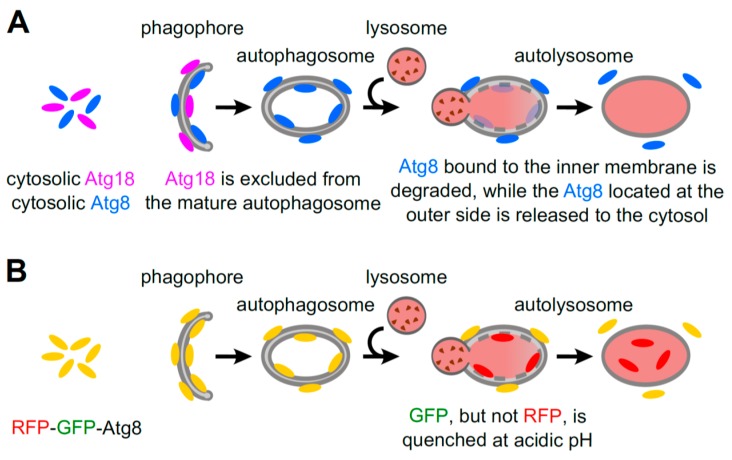
Atg18 and Atg8 as autophagic markers. (**A**) Atg18 is necessary for autophagosome formation, but it is released from mature autophagosomes. The cytosolic Atg8 protein is conjugated to phosphatidylethanolamine (PE) upon induction of autophagy, and is stably integrated into both the outer and the inner membranes of the phagophore. Therefore, the Atg8 fraction residing inside the autophagosome is degraded, together with other autophagic cargos, after lysosomal fusion; (**B**) red fluorescent protein (RFP)-GFP-Atg8 binds the autophagosomal membranes when autophagy is induced. Inside the autolysosomes, GFP is quenched due to the low pH, but RFP remains fluorescent. The increase in yellow and red Atg8 indicates induction of the autophagic flux, while the increase only in yellow Atg8 structures implies autophagic flux blockage.

Quantitative studies of the number of autophagic structures can be achieved by confocal microscopy of cells expressing a fusion of the green fluorescent protein (GFP) with Atg8 (microtubule-associated protein 1A/1B-light chain 3 (LC3) in mammals), which labels all stages of autophagosome formation, or Atg18 (WD-repeat protein interacting with phosphoinositides (WIPI) in mammals), which only marks the expanding phagophore [47]. When autophagy is induced, Atg8 is conjugated to phosphatidylethanolamine (PE) and is incorporated into the membrane of the expanding phagophore, where it remains until the autophagic body is degraded (Figure 4A). This marker has also been used extensively in *Dictyostelium* to monitor autophagosome formation and to study the dynamics of autophagosomes [2,37,39]. Using GFP-Atg8, it has been possible to perform in vivo time-lapse studies that followed the whole process from the formation to the closure of the autophagosome, and to establish the timing of this process. As autophagosomes move quickly, they are difficult to track for long periods of time, and thus it is advantageous to slightly compress the cells by overlaying them with a thin agarose sheet [2,39]. In addition, since GFP-Atg8 translocates to autophagosomal membranes during autophagy induction, one can monitor both the decrease in the fluorescence intensity of the cytosolic fraction and the concomitant increase in the number of GFP-Atg8 structures [2,39]. It is worth noting, however, that *Dictyostelium* possesses two Atg8 orthologues, which appear to play partly non-redundant roles in autophagosome formation, with different dynamics of recruitment [48,49].

Atg18 and its mammalian orthologues are essential for autophagosome biogenesis and recruited to the membranes of nascent and elongating autophagosomes via their phosphatidylinositol 3-phosphate (PtdIns(3)P) binding domain [50]. Therefore, GFP fusions of Atg18 allow the tracking of PtdIns(3)P-enriched membranes during autophagosome formation, and in contrast to Atg8, is lost immediately following closure. GFP-Atg18 has emerged as a robust marker after some difficulties using GFP-Atg8 were noticed. GFP-Atg8 has been observed to aggregate and localize to large punctate structures in *Dictyostelium* cells lacking autophagy, thereby causing aberrations [2,51], making this marker unsuitable for quantitative analysis in some experiments. In contrast, GFP-Atg18 does not aggregate, and its puncta can also be tracked and quantified. The small GFP-Atg18 puncta seem to expand into cup-like structures, resembling mammalian omegasomes [2,39]. Because Atg18 is released from mature autophagosomes in both *Dictyostelium* and mammalian cells, it only indicates the rate of autophagosome formation, and is not representative of the total number of autophagosomes in a cell. Therefore, to obtain a complete picture, it is advisable to monitor and quantitate both Atg8- and Atg18-positive structures. Additionally, as antibodies against the two Atg8 *Dictyostelium* orthologues have been generated, the endogenous proteins can be evaluated using immunohistochemistry [48,52].

## 4. Measurement of Gene and Protein Expression Levels

Although autophagy is essential for *Dictyostelium* development, the regulation of the expression of autophagy genes during development has not been studied in detail. The first descriptions were obtained using Northern blots to monitor the expression of *atg1*, *atg8*, *atg5*, *atg9*, and *atg12*, which all increase in mRNA level at early times during development [29]. An alternative, non-radioactive method is quantitative reverse transcription polymerase chain reaction qRT-PCR [39]. In addition, the steady-state protein levels of the two *Dictyostelium* Atg8 orthologues were recently analyzed by western blot, and were shown to have different expression patterns during development [48]. Unfortunately, while in mammalian cells it is possible to separate and quantify the cytoplasmic and autophagosome-associated lipidated forms of Atg8 (LC3 I/II), in *Dictyostelium* only a single form can be observed, and consequently Atg8 western blotting results must be interpreted carefully. It is possible that the fast-migrating lipidated form is under the detection level. Consequently, this technique can only shed light on the total amount of Atg8, and thus an increase in Atg8 levels can be interpreted as a rise in autophagy induction or as an autophagy blockage [36].

Gene and protein expression analyses can be useful as initial approaches to evaluate mutants or novel autophagy inducers, but cannot provide enough evidence of the real cellular autophagic activity, and so must be accompanied by other assays.

## 5. Autophagic Flux Assays

To perform a complete autophagy evaluation, it is important to analyze not only autophagosome formation, but also whether the cargos are being degraded. As discussed above, GFP-Atg8 puncta reflect only the steady-state levels of autophagosomes; therefore, the presence of higher number of puncta may be due either to an increase in autophagosome induction or to an autophagy blockage at a subsequent stage. In order to circumvent this problem, a number of autophagic flux assays have been developed in *Dictyostelium*.

### 5.1. Protein Cleavage Assays

**Figure 5:**
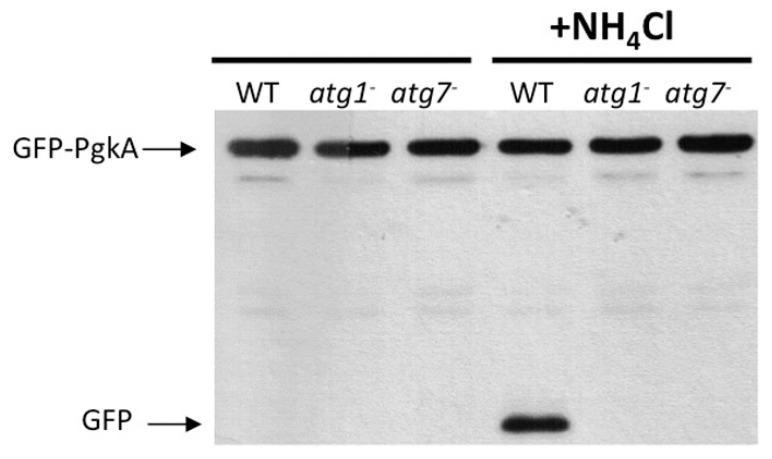
Protein cleavage assay. AX4 wild-type (WT), *atg1*^-^, or *atg7*^-^ cells constitutively expressing GFP-PgkA (phosphoglycerate kinase) were subjected to two 150 mM pulses of NH4Cl, each of 2 h, as previously described (see text). Total cell extracts were analyzed by western blot using an anti-GFP antibody. Free GFP cannot be detected in autophagy-deficient strains.

In many organisms, the autophagic flux is assayed by following the breakdown of a nominal cytoplasmic protein fused to the relatively hydrolase-resistant GFP. In *Dictyostelium*, this technique has been developed using either transketolase (Tkt) or phosphoglycerate kinase (PgkA) fused to GFP as cargoes [37,53]. As *Dictyostelium* possesses extremely acidic lysosomes with a pH of 3.5 or below [54], the use of non-saturating concentrations of the lysosomotropic agent NH_4_Cl is essential to visualize the free GFP fragments that accumulate from autophagic degradation (Figure 5). It is important to point out that the NH_4_Cl concentration required to visualize the GFP fragments is very high, inducing an elevated autophagic response per se that hinders the use of this assay to evaluate the effect of autophagy-inducing agents, although cells can be treated with other drugs prior to NH_4_Cl treatment [55]. In addition, NH_4_Cl also induces osmotic swelling of endosomes and lysosomes, which severely impairs microscopy observations. Nevertheless, this cleavage assay has proven to be very effective to evaluate the degradation capacity of both autophagy-deficient and over-activated strains [22,33,56]. This technique is very sensitive to changes in the metabolic status of the cells, so each experiment must be carefully controlled. For example, cells should have similar growth history, avoiding overconfluency. In addition, comparisons should always be performed within the same parental strain, since differences in the lysosomal pH and the autophagic capacity between the commonly used laboratory strains may occur. In this case, and when evaluating strains that present moderate phenotypes, it is advisable to optimize the NH_4_Cl concentration and/or compare the autophagic flux at different concentrations of NH_4_Cl within the same experiment.

An alternative technique, based on the breakdown of GFP-Atg8, does not require the use of NH_4_Cl. When the flux is completely blocked by, for instance, impairment of the lysosomal activity with a protease inhibitor treatment, the accumulation of GFP-Atg8 can be observed by western-blot while the free GFP disappears. On the other hand, when GFP-Atg8 is actively degraded, free GFP accumulates because of its relative resistance to degradation by lysosomal hydrolases. As this technique does not require the use of NH_4_Cl, it can be used to monitor autophagic changes upon drug or infection treatments [39]. Changes in the levels of free GFP are indicative of changes in the autophagic flux. However, interpretation of the results must take into account that both an induction or a blockage of autophagy may decrease the levels of free GFP, and additional flux assays are required to discriminate these possibilities [39]. In addition, it must also be considered that a small amount of free GFP is generated in an autophagy-independent manner as it occurs in the *atg1* mutant, which has been shown by other methods to have a complete block in autophagosome formation [32,39] (RE, personal communication).

### 5.2. Quantification of Fluorescently-Tagged Proteins

Imaging GFP-Atg8-expressing cells and counting GFP-Atg8 dots can also serve as a proxy readout for the autophagic flux if combined with inhibitors of degradation. In *Dictyostelium*, lysosomal inhibition can be achieved by treating the cells with a cocktail of protease inhibitors (Figure 2) or with the V-ATPase inhibitor concanamycin B [39]. Both the induction of autophagosome formation and the blockage of the autophagic flux lead to an increase in the number of GFP-Atg8 structures in the cell. Thus, when autophagy is induced, a higher number of GFP-Atg8 dots is expected when blocking the autophagic flux by the mentioned inhibitors. However, if the autophagic flux is already blocked, no change in the number of GFP-Atg8 structures will occur.

### 5.3. RFP-GFP-Atg8 Puncta Monitoring

Another kind of autophagic flux assay is based on the differential stability of green and red fluorescent proteins (GFP and RFP, respectively) in acidic environments. At a pH of 5 or below (which is found in lysosomes), the fluorescence of GFP is completely quenched, whilst RFP fluorescence is retained. For these assays, a fusion of Atg8 to a tandem GFP-RFP tag is expressed, and the autophagic flux is examined by monitoring the relative fluorescence of both tags with confocal microscopy. When green–red merged images of cells are analyzed, yellow puncta represent early autophagosomes, while red-only puncta (lacking green fluorescence) indicate acidic autolysosomes. Thus, when the autophagic flux is augmented, more of both yellow and red puncta are expected. However, when there is a blockage in lysosomal fusion or acidification, yellow puncta will accumulate (Figure 4B).

**Figure 6:**
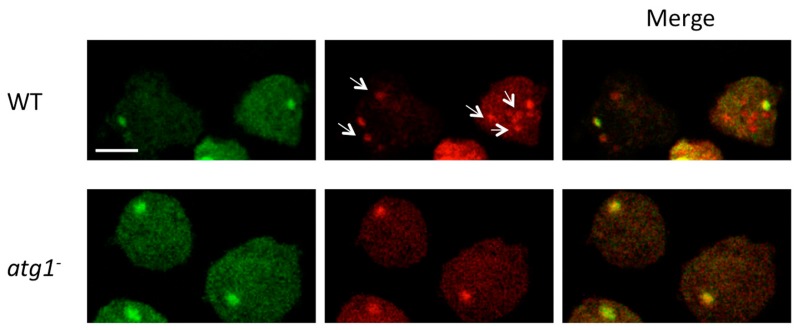
Wild-type (WT) (AX4) and *atg1*^-^ cells expressing the marker RFP-GFP-Atg8 were incubated for four hours with two pulses of 100 mM NH_4_Cl and visualized by confocal microscopy. The arrows show the presence of red-only puncta in WT that presumably represent autophagolysosomes. Cells lacking Atg1 typically show a large aggregate and no red-only puncta. Scale bar 5 μm.

The GFP-RFP-Atg8 construct has been evaluated in *Dictyostelium* [53], and it was observed that the use of NH_4_Cl is required to reveal the presence of autolysosomes (red-only puncta) in the strain AX4, likely because the extremely low lysosomal pH in this organism even quenches the fluorescence of RFP (Figure 6). In AX2, however, it has been reported that this technique can be applied without the need of NH_4_Cl, as red-only puncta are present under starvation [57].

## 6. Determination of Ubiquitin Aggregates

Autophagic dysfunction often leads to an abnormal increase of ubiquitinated protein aggregates. This is because either autophagy participates in the clearance of ubiquitinated proteins, or because in its absence the autophagy targets remain long enough to get ubiquitinated [58]. In either case, the increase in ubiquitin aggregates in autophagy-deficient strains has been exploited to evaluate the autophagic capacity of cells.

**Figure 7:**
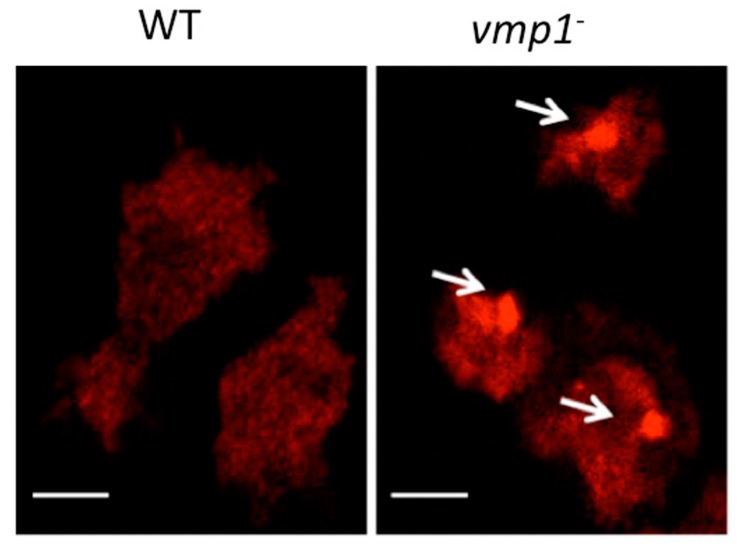
Detection of ubiquitinated protein aggregates by confocal microscopy. WT (AX4) and *vmp1*^−^ cells were fixed and prepared for immunocytochemistry detection of ubiquitin (red), and analyzed by confocal microscopy. Autophagy-deficient *vmp1*^−^ cells are not able to degrade proteins by autophagy and accumulate large ubiquitinated aggregates (denoted by white arrows). Scale bars 5 μm.

Ubiquitinated protein aggregates can be detected either by analyzing the Triton-insoluble fraction of total cellular lysates by western blot, or directly by the observation of immunohistochemically labeled cells by microscopy (Figure 7). In *Dictyostelium*, this technique revealed that in autophagy-deficient mutants the accumulation of ubiquitin aggregates correlates with impairment of the autophagic flux [36,37,51]. This method is thus useful to indirectly visualize impairments in autophagy, but quantitative analyses are difficult and should be complemented with other techniques. Note that defective proteasomal degradation can also cause an increase in ubiquitinated proteins.

## 7. Concluding Remarks

We have presented the current available strategies to assess autophagic activity in *Dictyostelium*. It is important to emphasize that—as in any other organism and cell type—there is no single perfect method to determine autophagic activity. The use of different strategies—even with overlapping approaches—should therefore be considered to accurately monitor autophagy in *Dictyostelium*. Additionally, due to the high conservation of this process, new methods can surely be adapted from other species to the study of autophagy in this model organism.
